# Acidic tumor microenvironment abrogates the efficacy of mTORC1 inhibitors

**DOI:** 10.1186/s12943-016-0562-y

**Published:** 2016-12-05

**Authors:** Seraina Faes, Adrian P. Duval, Anne Planche, Emilie Uldry, Tania Santoro, Catherine Pythoud, Jean-Christophe Stehle, Janine Horlbeck, Igor Letovanec, Nicolo Riggi, Nicolas Demartines, Olivier Dormond

**Affiliations:** 1 Lausanne University Hospital CHUV and University of Lausanne, Pavillon 4, Av. de Beaumont, 1011 Lausanne, Switzerland; 2 Mouse Pathology Facility, Lausanne University Hospital CHUV and University of Lausanne, Lausanne, Switzerland; 3 Institute of Pathology, Lausanne University Hospital CHUV and University of Lausanne, Lausanne, Switzerland; 4 Current Address: Swiss Institute of Experimental Cancer Research (ISREC), Swiss Federal Institute of Lausanne (EPFL), Lausanne, Switzerland

**Keywords:** Tumor Microenvironment, Acidity, mTORC1, Rapamycin, Sodium Bicarbonate, Resistance Mechanisms

## Abstract

**Background:**

Blocking the mechanistic target of rapamycin complex-1 (mTORC1) with chemical inhibitors such as rapamycin has shown limited clinical efficacy in cancer. The tumor microenvironment is characterized by an acidic pH which interferes with cancer therapies. The consequences of acidity on the anti-cancer efficacy of mTORC1 inhibitors have not been characterized and are thus the focus of our study.

**Methods:**

Cancer cell lines were treated with rapamycin in acidic or physiological conditions and cell proliferation was investigated. The effect of acidity on mTORC1 activity was determined by Western blot. The anticancer efficacy of rapamycin in combination with sodium bicarbonate to increase the intratumoral pH was tested in two different mouse models and compared to rapamycin treatment alone. Histological analysis was performed on tumor samples to evaluate proliferation, apoptosis and necrosis.

**Results:**

Exposing cancer cells to acidic pH in vitro significantly reduced the anti-proliferative effect of rapamycin. At the molecular level, acidity significantly decreased mTORC1 activity, suggesting that cancer cell proliferation is independent of mTORC1 in acidic conditions. In contrast, the activation of mitogen-activated protein kinase (MAPK) or AKT were not affected by acidity, and blocking MAPK or AKT with a chemical inhibitor maintained an anti-proliferative effect at low pH. In tumor mouse models, the use of sodium bicarbonate increased mTORC1 activity in cancer cells and potentiated the anti-cancer efficacy of rapamycin. Combining sodium bicarbonate with rapamycin resulted in increased tumor necrosis, increased cancer cell apoptosis and decreased cancer cell proliferation as compared to single treatment.

**Conclusions:**

Taken together, these results emphasize the inefficacy of mTORC1 inhibitors in acidic conditions. They further highlight the potential of combining sodium bicarbonate with mTORC1 inhibitors to improve their anti-tumoral efficacy.

## Background

Tumor cells preferentially perform glycolysis despite the presence of oxygen [1]. Consequently, an increased quantity of H^+^ is generated, creating a hostile environment characterized by acidic extracellular pH. In addition, tumors frequently present hypoxic regions due to insufficient blood supply, also promoting anaerobic metabolism and the formation of lactic acid [2]. Emerging evidence highlights that acidic tumor microenvironment not only promotes tumor progression, invasion and metastasis but also induces drug resistance [3–5]. Accordingly, therapeutic strategies that interfere with acid-base regulation have demonstrated anti-tumor activity in a variety of pre-clinical studies [6]. One of these strategies consists of oral administration of sodium bicarbonate in order to increase the intra-tumoral pH, resulting in an inhibition of tumor growth and metastasis formation in murine models [3, 7, 8]. Furthermore, sodium bicarbonate potentiates the efficacy of weak base chemotherapies such as doxorubicin presumably by enhancing drug uptake [5].

The complex 1 of the mechanistic target of rapamycin (mTORC1) represents a promising target in cancer therapies as it is frequently activated in cancer and as it controls cell growth [9, 10]. mTORC1 is composed of five different proteins: mTOR, Raptor, mLST8, PRAS40 and Deptor. The precise functions of mTORC1 components are still not fully characterized. Nevertheless, it was shown that Raptor positively regulates mTORC1 activity presumably by regulating the assembly of the complex and by recruiting substrates for mTOR [11, 12]. mTORC1 activity is regulated by a variety of stimuli. Whereas growth promoting factors induce mTORC1 activity, unfavorable growth conditions such as hypoxia or acidity generally lead to its inhibition [13, 14]. Once activated, mTORC1 regulates multiple cellular processes implicated in cell growth including protein, lipid and nucleotide synthesis [13]. Several studies have outlined the potential of inhibiting mTORC1 by rapamycin or its analogs termed rapalogs to reduce tumor progression in experimental models and to increase progression free survival in tumor patients [15, 16]. Unfortunately, similar to other targeted therapies, cancers relapse after an initial response to mTORC1 inhibition through the development of resistance mechanisms by cancer cells. Most identified resistance mechanisms involve the abolishment of negative feedback loops induced by mTORC1 inhibition, resulting in the activation of other proliferative signals [17–19]. In particular, loss of mTORC1/S6K1 mediated IRS-1, Grb10 and Sin-1 phosphorylation leads to aberrant overactivation of mTORC2/AKT signaling pathway which promotes tumorigenesis [19–21]. Therefore, therapeutic strategies overcoming these resistances against mTORC1 inhibitors need to be developed.

Although several resistance mechanisms to rapalogs, most of them implicated in intracellular processes, have been identified, little is known about the influence of acidic tumor microenvironment on the anti-cancer efficacy of these inhibitors. In the current work, we demonstrate that acidity reduces the antiproliferative effects of rapamycin in vitro and that sodium bicarbonate potentiates the anti-cancer activity of rapamycin in vivo. Thus, our findings identify the acidic tumor microenvironment as a novel parameter of resistance to mTORC1 inhibitors and provide a rationale to combine strategies that increase the intra-tumoral pH with mTORC1 inhibitors in cancer therapy.

## Methods

### Cell culture, reagents, antibodies

Human colorectal adenocarcinoma cell line HT29, human renal cell carcinoma cell lines 786–0 and Caki-1 as well as human hepatocellular carcinoma cell line PLC-PRF/5 were purchased from ATCC. Murine colon adenocarcinoma cell line MC-38 were kindly provided by Dr. Jeffrey Schlom (National Cancer Institute, NIH, Bethesda, MD, USA) [22]. Cell lines were cultured in Dulbecco’s Modified Eagle’s Medium-high glucose (DMEM) (Sigma-Aldrich) supplemented with 10% FBS and 1% streptomycin/penicillin. Rapamycin (#R-5000) and U0126 (#U-6770) were from LC Laboratories. MK-2206 was from Selleck Chemicals (#S1078). Sodium bicarbonate and HEPES (4-(2-hydroxyethyl)-1-piperazineethanesulfonic acid) were from Sigma-Aldrich. For cell culture, rapamycin was dissolved in dimethyl sulfoxide (DMSO). For in vivo experiments, rapamycin was dissolved in DMSO and diluted 1:5 in PBS-Tween-PEG (89.6% phosphate-buffered saline (PBS), 5.2% Tween 20, 5.2% poly (ethylene glycol)). For immunohistochemical staining, the following primary antibodies and concentrations were used: anti-phospho S6 ribosomal protein antibody (1:100) (M3500; Spring Bioscience Corporation), anti-PCNA antibody (1:50) (ab2426; Abcam) and anti-cleaved Caspase-3 antibody (1:200) (#9661; Cell Signaling Technology). For Western blot analysis, the following primary antibodies and concentrations were used: Anti-phospho S6 ribosomal protein antibody (1:2000) (M3500; Spring Bioscience Corporation), anti-S6 ribosomal protein antibody (1:1000) (#2217; Cell Signaling Technology), anti-raptor antibody (1:1000) (#2280; Cell Signaling Technology), anti-phospho-Akt antibody (1:500) (#4060; Cell Signaling Technology), anti-Akt antibody (1:1000) (#2920; Cell Signaling Technology), anti-p44/42 MAPK antibody (1:1000) (#9102; Cell Signaling Technology), anti-phospho-p44/42 MAPK antibody (1:1000) (#9101; Cell Signaling Technology), anti-phospho-4E-BP1 (Thr37/46) antibody (1:1000) (#2855; Cell Signaling Technology), anti-phospho-4E-BP1 (Ser65) antibody (1:1000) (#9451; Cell Signaling Technology), anti-4E-BP1 antibody (1:1000) (#9644; Cell Signaling Technology) and anti-actin antibody (1:5000) (#A2228; Sigma Aldrich).

### Proliferation assay

Cancer cells were plated in 96 well plates at 10 000 cells per well, cultured in DMEM adjusted to different pH using HEPES and treated with DMSO, rapamycin (100 nM), MK-2206 (1 μM) or UO126 (10 μM) for 48 h. Cellular proliferation was monitored after 48 h with CellTiter 96 AQ_ueous_ One Solution Cell Proliferation Assay (MTS) (Promega) by following the manufacturer’s instructions. Absorbance at 492nm was measured 30 min after compound addition. Experiment was performed in quadruplicates and repeated three times.

### Stable transfection

Lentiviruses were generated by transfecting HEK-293T cells with the following plasmids: psPAX2 (plasmid #12260, Addgene) and pMD2.G (plasmid #12259, Addgene) together with raptor_1 shRNA (plasmid #1857, Addgene) or a control shRNA (plasmid #1864, Addgene) using FuGENE and following the manufacturer’s instructions [23]. Supernatants were collected and used to infect HT29 cells. Stable transfectants were selected for resistance to puromycin (10 μg/ml). Efficiency of raptor downregulation was tested by Western blot.

### Western blot analysis

Cell lines were plated in 6 well plates at 100 000 cells per well and cultured in DMEM adjusted to different pH using HEPES. Cells were cultured at different pH for different times and treated with rapamycin 100 nM or DMSO as indicated. Cells were lysed in RIPA buffer. Protein concentrations were measured using BCA Assay (Pierce). Equal amounts of protein (20 μg) were separated on 4–12% polyacrylamide gel and subsequently transferred to a polyvinylidene difluoride membrane (Millipore). Membranes were blocked with Odyssey blocking buffer (LI-COR Biosciences) and immunoblotted with primary antibodies followed by infrared secondary antibodies. Bands from immunoreactive proteins were visualized by an Odyssey infrared imaging system (LI-COR Biosciences).

### Immunohistochemistry

Xeno- and allografts were fixed in 4% formaline overnight, dehydrated with ethanol and paraffin-embedded. Sections of 3 μm were obtained using MICROM HM 355S microtome (Thermo Scientific), and tissue sections were mounted on Superfrost Plus slides (Thermo Scientific). Slides were then deparaffinized and rehydrated with xylol and alcohol. After antigen retrieval (citrate pH 6.0 or TRIS/EDTA pH 9.0), sections were immunostained using above-mentioned primary antibodies for 60 min and subsequently incubated with Dako EnVision HRP secondary antibody (Dako) for 30 min. Counterstaining of nuclei and controls with secondary antibodies only were performed. In parallel, staining with haematoxylin and eosin were performed. One section from each xenograft and allograft tumor and three tumors for each condition were analyzed for each staining. Carl Zeiss Axioscope, AxioCam MRc and AxioVision 40V 4.6.3.0 software (Carl Zeiss Vision) were used for imaging acquisition and processing. Histology analysis was performed by two researchers blinded to groupings. Percentage of tumor necrosis (light pink stained surface in H & E) and phospho S6 expression (phospho S6 positive surface) were measured quantitatively using ImageJ 1.46r Threshold Colour Plugin by analyzing 10 representative images of 3368 × 2668 μm for each condition in three different tumors. PCNA positive and PCNA negative cancer cells and cleaved caspase 3 positive and cleaved caspase 3 negative cancer cells respectively were counted in 10 representative vital tumor zones of a 100 × 100 μm surface for three different HT29 and MC-38 tumors. Percentage of PCNA positive and cleaved caspase 3 positive cells were calculated by dividing the number of PCNA or cleaved caspase 3 positive cancer cells by the number of PCNA or cleaved caspase 3 positive and negative cancer cells respectively.

### Mouse models

Animal experiments were in accordance with the Swiss federal animal regulations and approved by the local veterinary office. Female nude and C57BL/6 eight-week old mice were purchased from Janvier Labs. Mice were randomized into different groups (*n* = 5/group; groups “vehicle” - “bicarbonate” - “rapamycin” - “bicarbonate and rapamycin”). HT29 (3 × 10^6^) and MC-38 (1 × 10^6^) cells were injected subcutaneously into the right flank. Sodium bicarbonate was added to the drinking water at a concentration of 200 mmol/L, starting 1 day before cancer cell injection. Once the tumor xeno-/allografts reached a mean volume of 25 mm^3^, mice were treated once daily with rapamycin (3 mg/kg body weight, intraperitoneally, in 20 μl DMSO and 80 μl PBS-Tween-PEG) or vehicle (20 μl DMSO and 80 μl PBS-Tween-PEG). Tumor volumes were measured daily using a caliper and calculated with the formula V = A * B * C * π/6 where A is the length, B the width and C the height of the tumor. Animals were sacrificed once the biggest tumor of vehicle treated mice reached the size of 1 000 mm^3^ (defined as interruption criterion according to veterinary recommendations). Tumors were excised and samples processed for immunohistochemical analysis.

### Statistics

Statistical analysis including Student’s *t*-test, One-way ANOVA and Two-way ANOVA were carried out as appropriate using GraphPad Prism version 6.05.

## Results

### mTORC1 inhibition does not reduce cancer cell proliferation in acidic conditions

**Fig. 1:**
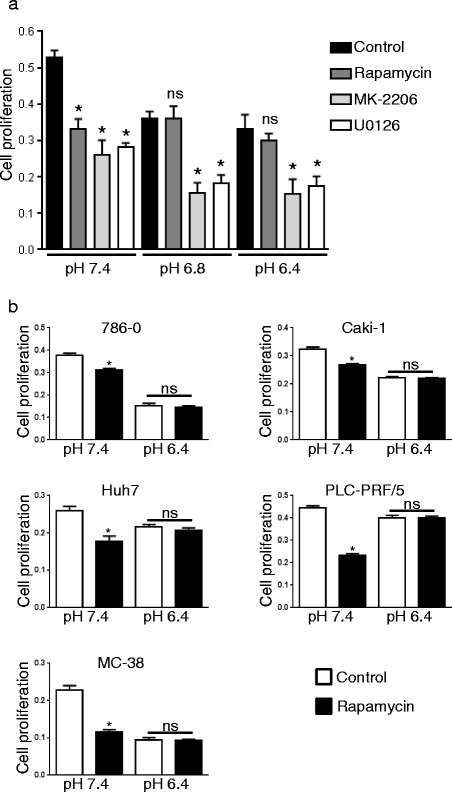
Acidic extracellular pH reduces the antiproliferative efficacy of rapamycin. **a** HT29 cells were cultured in medium buffered to pH 7.4, 6.8 or 6.4 and treated with rapamycin (100 nM), MK-2206 (1 μM), U0126 (10 μM) or DMSO for control. MTS proliferation assay was performed after 48 h of treatment. Bar charts represent mean, error bars represent SD. * *p* < 0.05, Student’s *t* test compared to control cells at the same pH. **b** 786–0, Caki-1, Huh7, PLC-PRF/5 and MC-38 cells were cultured at pH 7.4 or 6.4 and treated with rapamycin (100 nM) or DMSO for control. MTS proliferation assay was performed after 48 h of treatment. Bar charts represent mean, error bars represent SD. * *p* < 0.05, Student’s *t* test compared to control cells at the same pH

We first investigated whether extracellular pH influences the antiproliferative efficacy of rapamycin. To test this, human HT29 colon cancer cells were cultured under various pH conditions and treated with rapamycin. Cell proliferation was monitored after 48 h of treatment. Rapamycin significantly reduced cancer cell proliferation by 37.1% (*p* < 0.0001) under physiological pH. This effect was however lost when cancer cells where cultured at pH 6.8 or 6.4 (Fig. 1a). For comparison, we treated HT29 cells with MK-2206, a chemical inhibitor of AKT, and U0126, a chemical inhibitor of mitogen-activated protein kinase kinase 1/2 (MEK1/2). In contrast to rapamycin treatment, MK-2206 and U0126 still significantly reduced cell proliferation in acidic conditions (Fig. 1a). The loss of efficacy of rapamycin in acidic conditions was not restricted to HT29 cells as we found similar results with a larger panel of cancer cells including human renal carcinoma cell lines (786–0, Caki-1), human hepatocellular cancer cell lines (Huh7, PLC-PRF/5) and murine colon adenocarcinoma cell line (MC-38) (Fig. 1b).

**Fig. 2:**
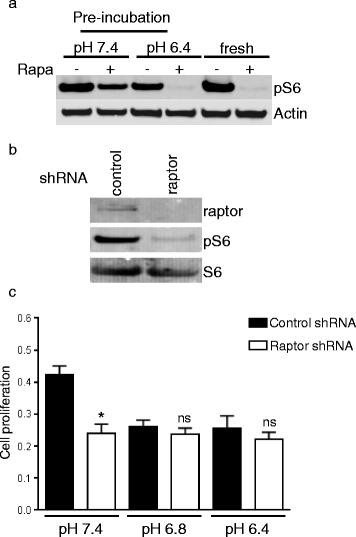
Acidity does not inactivate rapamycin. **a** HT29 cells were treated with rapamycin (100 nM) that was previously incubated in DMEM full medium buffered at pH 7.4 or 6.4 for 24 h. As a control HT29 cells were also treated with fresh rapamycin. After 3 h of treatment, cells were lysed and lysates analyzed by Western blot. **b** HT29 cells were infected with lentiviruses expressing a control or raptor shRNA. Following selection, cancer cells were lysed and Western blot analysis was performed with the indicated antibodies. **c** HT29 cells generated in panel **b** were exposed to various pH and proliferation assay was carried out after 48 h of exposition. Bar charts represent mean, error bars represent SD. * *p* < 0.0001, ns = not significant, Student’s *t* test

To exclude a loss of rapamycin efficacy by inactivation in acidic conditions we performed the following experiment. Rapamycin was incubated for 24 h at a concentration of 1 μM in medium buffered at pH 6.4 or 7.4. DMSO diluted in medium at the same dilution was used as control. Subsequently, medium was collected, diluted in medium of pH 7.4 at a concentration of 1:10 (for a final rapamycin concentration of 100 nM) and transferred on HT29 cells. The ability of rapamycin to block mTORC1 was assessed after 24 h by Western blot using phosphorylation of S6 ribosomal protein as a read-out of mTORC1 activity. We found that rapamycin previously exposed to acidic pH still significantly decreased S6 phosphorylation (Fig. 2a). In comparison, the efficacy of rapamycin exposed to pH 7.4 for the entire 48 h time period was reduced, suggesting that acidity does not inactivate rapamycin.

In order to further substantiate that mTORC1 inhibition does not reduce cancer cell proliferation in acidic conditions, we used a lentiviral short hairpin RNA (shRNA) expressing system that downregulates the expression of raptor and hence blocks the activity of mTORC1. Western blot analysis confirmed the reduced expression of raptor as well as the inhibition of mTORC1 as evidenced by the lack of S6 ribosomal protein phosphorylation (Fig. 2b). Similarly to what we observed with rapamycin, downregulation of raptor reduced cancer cell proliferation by 43.8% (*p* < 0.0001) at physiological pH but did not result in a significant antiproliferative effect when medium was buffered to pH 6.8 or 6.4 (Fig. 2c). Taken together, these results suggest that blocking mTORC1 in acidic conditions does not reduce cancer cell proliferation.

### mTORC1 is inhibited by acidity

**Fig. 3:**
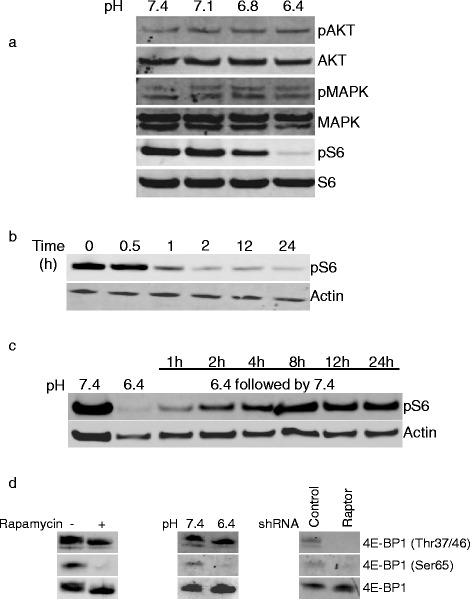
Reversible inhibition of mTORC1 by acidic pH. **a** HT29 cells were cultured for 3 h in medium buffered at the indicated pH values. Cells were subsequently lysed and Western blot analysis was performed with the indicated antibodies. **b** HT29 cells were exposed to medium buffered at pH 6.4 for the indicated time periods. Cell lysates were prepared and analyzed by Western blot for phospho S6 ribosomal protein and actin expression. **c** HT29 cells were cultured at pH 6.4 for 3 h followed by restoration of pH to 7.4 for the indicated time periods. Subsequently, cell lysates were prepared and analyzed by Western blot. **d** Western blot analysis of lysates prepared from HT29 cells treated for 3 h with rapamycin (100 nM) or DMSO as a control (*left panel*), HT29 cells exposed for 3 h to medium buffered to pH 7.4 or 6.4 (*middle panel*), or HT29 cells expressing a control or a raptor shRNA (*right panel*)

We next determined the influence of acidic pH on mTORC1 activity. HT29 cells were exposed to medium buffered at pH ranging from 7.4 to 6.4 and mTORC1 activity was analyzed by Western blot. We observed a reduction of mTORC1 activity in acidic conditions as evidenced by a decreased phosphorylation of S6 ribosomal protein (Fig. 3a). This reduction was already present at pH 6.8 and maximal at pH 6.4. In contrast, AKT or MAPK phosphorylation was not affected by acidic pH (Fig. 3a). The reduction of mTORC1 activity by acidic pH started already after 60 min exposure to acidity and was completely reversible (Fig. 3b-c).

Recent studies have demonstrated that some functions of mTORC1 are not inhibited by rapamycin [24]. For instance, rapamycin only blocks the phosphorylation of Ser65 but not Thr37/47 of 4E-BP1, although all these residues are phosphorylated by mTORC1. We therefore tested whether acidic pH inhibits mTORC1 completely or only the rapamycin sensitive functions of mTORC1. We observed that, similarly to rapamycin, acidic pH reduced pSer65 4E-BP1 but not pThr37/47 4E-BP1 as shown by Western blot (Fig. 3d). In contrast, the inhibition of mTORC1 using interfering shRNA to raptor blocked both Ser65 and Thr37/47 phosphorylation. Taken together, these results illustrate that acidic pH specifically inhibits the rapamycin sensitive functions of mTORC1.

### Sodium bicarbonate potentiates the anti-tumor efficacy of rapamycin in vivo

**Fig. 4:**
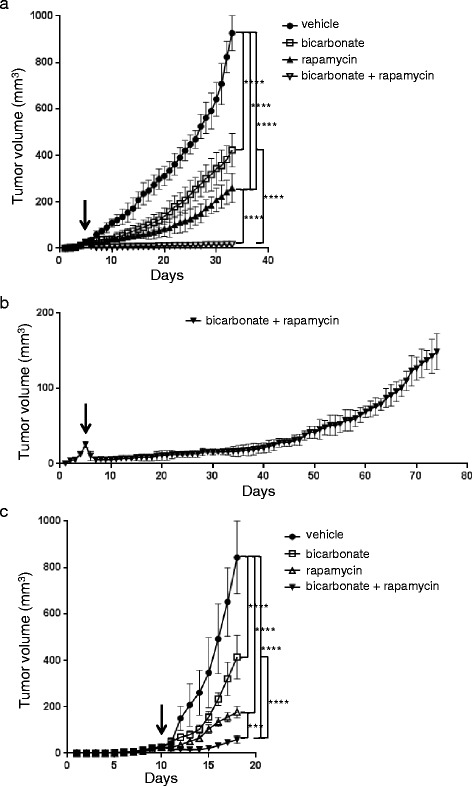
Sodium bicarbonate potentiates the anticancer efficacy of rapamycin. **a** HT29 xenograft growth curves treated with vehicle, sodium bicarbonate (200 mmol/L drinking water), rapamycin (3 mg/kg i.p. per day) or a combination of both. **b** HT29 xenograft growth curve treated with sodium bicarbonate (200 mmol/L drinking water) and rapamycin (3 mg/kg i.p. per day). **c** MC-38 allograft growth curves treated as under **a**. Arrows denote the start of treatment with rapamycin and vehicle at a mean graft volume of 25 mm^3^. **** *p* < 0.0001, *** *p* < 0.001, *n* = 5/group, Two-way ANOVA

**Fig. 5:**
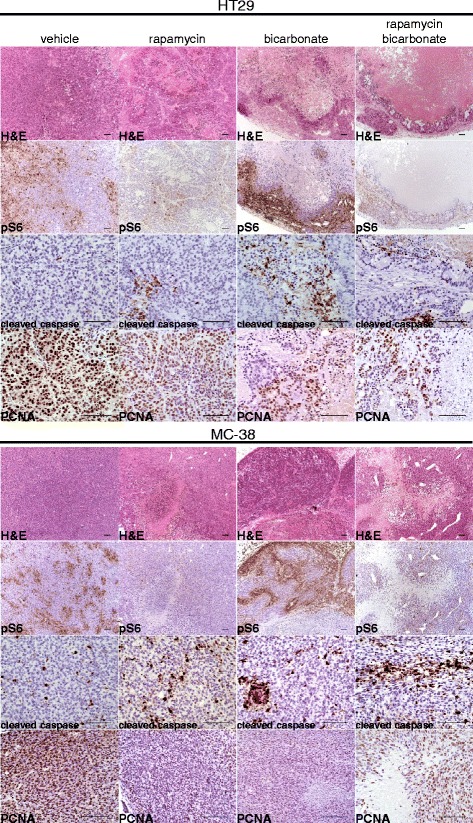
Bicarbonate induces mTORC1 activity, increases necrosis and apoptosis and potentiates rapamycin efficacy. Serial sections of HT29 tumor xenografts and MC-38 tumor allografts were stained with H & E and with phospho S6, cleaved caspase 3 and PCNA antibodies. Representative images of immunohistochemistry are shown. Scale bar, 100 μm

**Fig. 6:**
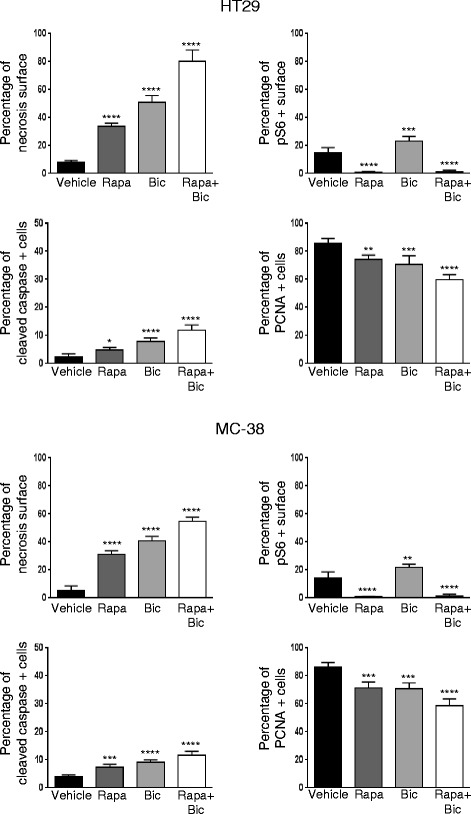
Effects of rapamycin and bicarbonate on tumor necrosis, mTORC1 activity, tumor apoptosis and proliferation. Percentage of tumor necrosis (*light pink* stained surface in H & E shown in Fig. 5) and phospho S6 expression (phospho S6 positive surface shown in Fig. 5) were compared for vehicle, sodium bicarbonate, rapamycin, and combined treatment in HT29 xenografts and MC-38 allografts in 10 representative sections of 3368 × 2668 μm for three different tumors using ImageJ Threshold Colour Plugin analysis. Percentage of PCNA positive cancer cells and percentage of cleaved caspase 3 positive cancer cells was counted in 10 representative zones of 100 × 100 μm for three different HT29 and MC-38 tumors. Bar charts represent mean, error bars represent SD. **** *p* < 0.0001, *** *p* < 0.001, ** *p* < 0.01, * *p* < 0.05, ns = not significant, One-way ANOVA

According to our in vitro observations, acidity impedes the anti-proliferative efficacy of rapamycin. Since the tumor microenvironment is typically acid compared to normal tissue, we next hypothesized that strategies aiming to increase the intratumoral pH might potentiate the anti-cancer efficacy of rapamycin. Recent studies have shown that the intratumoral pH of tumors grown in mice can be safely increased by treating mice with sodium bicarbonate [8]. Hence, to test our hypothesis, nude mice bearing HT29 tumor xenografts were randomized into control, rapamycin, sodium bicarbonate or rapamycin plus sodium bicarbonate groups. We saw that both rapamycin (growth inhibition by 71.9%) and sodium bicarbonate (growth inhibition by 54.4%) slowed the growth of HT29 tumor xenografts. Combininig rapamycin with sodium bicarbonate provided a stronger anti-cancer efficacy (growth inhibition by 98.3%) than either therapy alone (Fig. 4a). The effect was long lasting, as after 70 days of treatment, the volume of the tumor xenografts did still not exceed 150 mm^3^ (Fig. 4b). The superior anti-cancer efficacy of a combination of rapamycin and sodium bicarbonate could further be demonstrated in C57BL/6 mice bearing MC-38 tumor allografts (Fig. 4c). In both models, HT29 xenografts and MC-38 allografts, histological analysis showed that sodium bicarbonate enhances mTORC1 activity in tumor cells as evidenced by increased phospho S6 immunostaining (57.8% increase in HT29 and 52.9% in MC-38) (Figs. 5 and 6). Sodium bicarbonate also markedly increased tumor necrosis (6-fold increase in HT29 and 7.7-fold increase in MC-38), and the necrotic tumor surface was even more pronounced after a combined treatment of sodium bicarbonate and rapamycin (9.6-fold increase in HT29 and 10.3-fold increase in MC-38) (Figs. 5 and 6). Finally, combining sodium bicarbonate with rapamycin resulted in increased cancer cell apoptosis and reduced cancer cell proliferation as compared to either treatment alone (Figs. 5 and 6).

## Discussion

Even though targeting signaling pathways that are deregulated in cancer has shown efficacy in cancer therapy, presently, most therapies fail to cure patients. In fact, several impediments lessen the efficacy of these strategies, including genetic heterogeneity and resistance mechanisms [25]. In addition, the physical and chemical properties of the tumor microenvironment were shown to profoundly influence tumor biology and response to treatment. In this context, several studies support the anti-cancer strategy of targeting the acidic pH of a tumor [6, 26–28]. In the present study, we further substantiate that alkalinization of tumor pH enhances the anti-tumor efficacy of rapamycin and thus represents a valuable adjunct to mTORC1 inhibitors. Indeed, in accordance with others, we show that acidic pH downregulates mTORC1 activity [14]. We further demonstrate that, in acidic conditions, blocking mTORC1 with rapamycin or by genetic manipulation does not prevent cancer cell proliferation. This suggests that mTORC1 participates in cancer cell proliferation only at physiological pH and tumor cells proliferate independently of mTORC1 in an acidic tumor microenvironment. Indeed, sodium bicarbonate treatment restores mTORC1 activity in cancer cells and a subsequent inhibition of mTORC1 allows to further reduce cancer cell proliferation (Fig. 5). Of note, we cannot exclude that sodium bicarbonate also affect rapamycin metabolism, leading to a better uptake by cancer cells, resulting in a stronger inhibition of mTORC1.

One obvious explanation for the lack of efficacy of rapamycin in acidic conditions is its inactivation by acidity. However, we found that the biological activity of rapamycin is still present following incubation of rapamycin in acidic conditions (Fig. 2a). The inhibition of mTORC1 was even more pronounced when rapamycin was pre-exposed to acidity compared to a physiological pH (Fig. 2a). Consistent with our observation, it was reported that rapamycin is more stable in acidic conditions [29].

Besides acidity, tumor hypoxia has also been shown to negatively regulate mTORC1 activity [13]. Accordingly, mTORC1 activity is mainly restricted to the non-hypoxic tumor compartment, and the hypoxic tumor response mediated by HIF-1 induces resistance to mTORC1 inhibitors [30]. As hypoxia promotes tumor acidosis, a complex relationship exists between mTORC1 activity, hypoxia and acidity in tumors [2]. In addition, acidosis affects the hypoxic tumor response by increasing HIF-1 stability [2]. Therefore, targeting tumor acidosis might also reduce HIF-1-mediated cancer cell responses and by this mechanism enhance the anti-cancer efficacy of mTORC1 inhibitors. Clearly, additional studies are needed to further characterize the molecular relationship between mTORC1, hypoxia and acidity in cancer.

A simple approach to increase tumor pH has been proposed through the use of systemic buffers such as sodium bicarbonate [7]. Interestingly, this approach has demonstrated remarkable anti-cancer efficacy in mouse models. Sodium bicarbonate is sufficient to reduce tumor growth, local invasion and metastasis in preclinical models [3, 8]. Furthermore, it also prevents the de novo-formation of prostate cancer in a mouse model of spontaneous tumor formation [31]. Our findings further suggest that sodium bicarbonate might be effective as an adjunct to mTORC1 targeting therapies in order to potentiate their efficacy. Whereas the use of sodium bicarbonate in humans over a long period of time may induce serious side effects, a more restricted application such as an adjuvant setting might be better tolerated. Nevertheless, it is worth noting that long term therapy with sodium bicarbonate has been reported in patients with renal tubular acidosis and sickle cell anemia without major side effects [32, 33]. Its feasibility in the context of cancer needs, however, to be investigated. Of note, besides sodium bicarbonate, other methods that alter tumor pH, including proton pump inhibitors, were shown to exert anti-cancer activity and thus represent a therapeutic alternative, possibly better tolerated than sodium bicarbonate [34, 35].

The mechanisms underlying the anti-tumor effects of sodium bicarbonate need to be fully identified [36]. Here we observe that sodium bicarbonate significantly increases tumor necrosis, evoking a powerful inflammatory response (Fig. 5). This suggests that pH buffering by sodium bicarbonate influences the behavior of non-tumor cells present in the tumor microenvironment. Consistent with this hypothesis, acidity was shown to induce an M2-like polarization of tumor associated macrophages which promote tumor growth [37]. Furthermore, acidity also induces anergy of tumor-infiltrating T lymphocytes [38]. Hence, targeting tumor acidity might also represent an adjunct therapy to strategies aiming to modulate the immune response against tumor cells. Consistent with this hypothesis, a recent study showed that neutralizing tumor acidity with sodium bicarbonate improved the antitumor efficacy of anti-CTLA-4 or anti PD-1 therapies as well as of adoptive T cell transfer [39].

A discrepancy exists between our in vitro and in vivo results. Indeed, whereas alkaline conditions promote cancer cell proliferation in culture, it reduces tumor growth in nude mice. One possible explanation is that alkaline conditions favor an anti-tumor response by the tumor microenvironment. Consistent with this hypothesis, acidity promotes a tumor promoting phenotype of macrophages [37]. Similarly, the activity of natural killer cells is reduced in acidic conditions [40]. Further studies are however needed to fully characterize the consequences of targeting tumor acidity on the tumor micorenvironement.

## Conclusions

The present study shows that acidity acts as a novel resistance mechanism to mTORC1 inhibitors. In this regard, pharmacological interventions targeting tumor pH represent a therapeutic strategy to potentiate the anti-tumor efficacy of mTORC1 inhibitors; a new therapeutic approach that warrants clinical evaluation.

## Abbreviations

MAPK: Mitogen-activated protein kinase
mTORC1: Mammalian target of rapamycin complex 1
